# Sponge City Planning and Information System Development Based on Geographic Information Fuzzy Processing

**DOI:** 10.1155/2022/9464785

**Published:** 2022-09-15

**Authors:** Mingxin Gan, Tongfang Li

**Affiliations:** University of Science and Technology Beijing, Beijing 100083, China

## Abstract

During the development of the urban water system from 1.0 to 3.0, the impervious surface area gradually increased, hindering the natural infiltration and self-purification process of urban rainwater, resulting in serious urban water pollution, urban waterlogging in the rainy season, and groundwater problems. Since water will seep into the ground, serious water pollution will cause damage to the ground. Therefore, in dealing with urban rainwater problems, we want to use the sponge's ability to absorb and store water to build our cities into sponge cities. In this paper, we have constructed a sponge city planning and information system development based on geographic information fuzzy processing. We use differentiated fuzzy processing methods to eliminate classified information to achieve a perfect combination with nearby images and use ordinary fuzzy processing methods to solve the problem of nonconfidential information. This paper discusses the impact of natural topography on the planning and construction of sponge cities, including whether natural topography will affect rainwater, whether it will affect the distribution of different strata, and whether it will affect the utilization of groundwater resources. The basic functions of this platform are provided by a series of functions of GIS, and multiple modules are developed according to management requirements. The initial state of street view data is a lot of fisheye lens photos and corresponding point location information, which are displayed online after data preprocessing, detection information, editing information, and blurring processing.

## 1. Introduction

The stable development of a city depends on the city's infrastructure, strengthening people's awareness of environmental protection, improving the overall capacity of the city, improving city operation efficiency, and promoting urbanization [1]. In the conference on urban facility construction a few years ago, many people expressed some real opinions in this conference and proposed that my country's urban infrastructure construction should take the path of “green” development and proposed for the future development of my country Reasonable suggestion [2]. It is believed that the construction of sponge cities is an important turning point in the construction of urban infrastructure in my country. The earliest use of the term “sponge city” in my country comes from the method of using sponge soil to store water and fertilizer proposed by Chinese experts in the 20th century [3]. In 2010, some researchers proposed to transform the city into a sponge city, storing rainwater for later reuse during heavy rains, using rainwater for flood control and cooling on hot days, and using nature to give us resources for repeated use [4]. In 2011, researchers proposed that the “ecological sponge” area should be able to store rainwater like a sponge to facilitate subsequent use so that the rainwater can be fully utilized [5]. In 2012, another researcher proposed that the construction of a “sponge city” means that rainwater is directly discharged into a storage tank, the use of soil can seep and other reasons, combined with the recycling of rainwater and presenting it to people for visual enjoyment, to prevent urban construction from affecting the water cycle, etc., [6].

## 2. Methods of Fuzzy Processing of Geographic Information

### 2.1. Overall Platform Design

**Figure 1:**
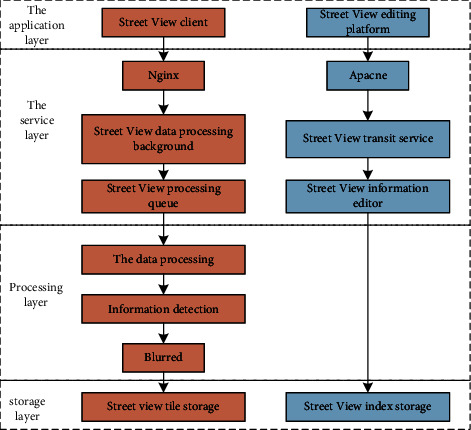
Structure design of street view security and confidential information processing platform.

The platform is controlled by a distributed storage computing system, which includes important modules such as data processing, detection information, editing information, and fuzzy processing. The structure design is shown in Figure 1.

**Figure 2:**
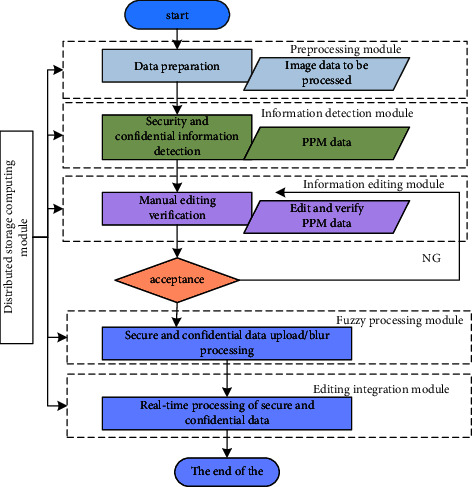
Platform function modules and processing flowchart.

The software platform composed of millions of servers handles millions of data every day. The average daily input data is millions, and the total number of images generated is billions. The platform is still running in an orderly manner and the processing results are accurate. The rate is quite high, which shows that the efficiency of this module is relatively good. In addition, the module can be easily expanded horizontally to double the calculation of business data to meet the needs of larger data calculations. The initial state of street view data is a lot of fisheye lens photos and corresponding point position information, which are displayed online after data preprocessing, detection information, editing information, and blur processing. The platform function module and processing procedure are shown as in Figure 2. The first data processing refers to the screening and sorting of relevant data before relevant work is carried out. This module will work day and night and can execute commands issued by different aspects, without interfering with each other. The detection information combines the automatic identification system of the computer software with the human operating system to achieve precise identification and selection of street views with strict security information. The location information provided by it is used to reverse the calculation of the information that is at risk of leakage. This process includes identifying pictures and associated topology. Editing information refers to the identification and screening of the identified security and confidential information based on the information detection results when the information detection results do not meet the requirements. If the judgment is wrong or the screening is unqualified, it can be re-edited through manual intervention Information, to achieve the final result we get is true and reliable and can withstand scrutiny data. Blur processing refers to the use of image blurring and texture transplantation to hide information that should not appear and find its location. There are different ways to process different types of data. For example, for processing private information, the platform uses Gaussian filters to filter the privacy area, and autonomously determines the degree of blurriness of the processed area, making it difficult for people to see the processed area. When processing nonpublic information, the platform perfectly integrates the surrounding information with the processed information, instead of deleting it directly.

NG transplantation coding makes the whole area look more consistent, without any sense of violation, and it is difficult to identify whether it has been processed or not. Editing integration will use two technical means to make work, approval, and online work together to perform high-efficiency work. Offline editing software uses the face to realize the automatic recognition process, assisting the manual more efficient detection and modification to obtain the final result. To achieve the highest editing efficiency. The online editing software can realize the entire process of manually correcting results and updating and processing a series of problems feedback from users within 30 minutes, and can flexibly handle various situations encountered. There is no influence between the data of each operating environment, and the results of editing can be shared at any time and any place, which maximizes the sharing of results and the data is also safe.

### 2.2. Key Technology Analysis

Internet Street View security and confidential information is widely distributed, has large differences, and does not have certain rules. There are the following difficulties in dealing with these problems: first, some information without obvious characteristics cannot be automatically extracted and identified; second, there are a small amount of information has similarities with nonconfidential security information, so processing with this method may lead to some wrong judgments. After processing, it is particularly easy to cause damage to the overall perception of the image, thereby affecting the product experience; third, although part Information can be distinguished from nonsecure and confidential information, but his algorithm cannot be applied to other types of data, and plays a very small role for a large amount of street view data; fourth, there may be a variety of information types in reality. But we can't build a complete recognition sample library to distinguish effectively; finally, under the condition of not affecting the judgment errors and omissions of classified information, both the integrity of the scene elements and the visual the aesthetics on the Internet has higher requirements for the security and confidential information processing technology of Internet Street View. In order to meet this requirement, we will use different methods to identify classified information and nonconfidential information. The differentiated fuzzy processing method eliminates confidential information and achieves a perfect combination with nearby images. The ordinary fuzzy processing method is used to solve the problem of nonconfidential information and protect personal privacy. Associated topology refers to some important location information provided to us through the information database when processing some undisclosed information data, rechecking and verifying the problems in the data, and finally manually confirming the elements and related secrets. The basis for the realization of the above technology is the establishment of street view data security and a confidential information database. Through nearly two years of collecting and processing street view data, Tencent has street view data for nearly 300 cities in China. In the current related scenes, the accuracy of detecting confidential information is quite high. The implementation of this technology determines the scope of the subsequent inspection data to maximize the efficiency of information inspection.

## 3. Sponge City Planning Strategy Based on Fuzzy Processing of Geographic Information

### 3.1. Data Acquisition and Preprocessing

In the past few years, the country proposed that by this year, most cities in the country have basically completed sponge construction. The construction of sponge cities mainly manages and controls urban construction, and systematically deals with the problem of interception of urban rainwater [7]. Through the above-given understanding and analysis, it is easy to know that terrain, climate factors, and rainfall conditions play an important role in the construction of sponge cities [8]. We must balance and coordinate the study of the geomorphic features of the regional climate, and use ArcGIS, SWMM, and other hydrological analysis simulation software to calculate and analyze the data to put forward reasonable suggestions. The important idea of this article is how to conform to nature so that nature can absorb, store and purify rainwater by itself. DLR is the DEM data obtained in the radar mapping method used in previous years [9]. DLR data are relatively high-precision data. Although this type of data has been covered in all aspects, it has not yet covered all areas. The main reason is that during the radar surveying and mapping process in 2000, German surveyors used Endeavour to measure and used X-band coverage, but the coverage area was not large. The Americans used C-band radar for coverage, and the Space Shuttle was based on C-band [10, 11]. The surveying and mapping require flying, resulting in a smaller area covered by the x-band.

**Figure 3:**
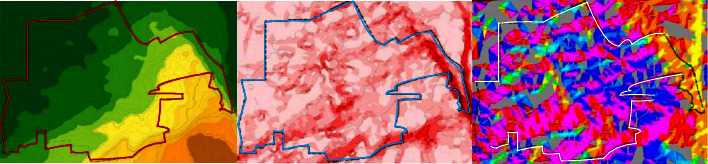
Analysis results of elevation, slope, and aspect of a city.

The high accuracy of DLR and GDEM-V2 data meets all the requirements for building a sponge city. In China, these two types of data need to be used in many places, but it is relatively difficult to obtain these two types of data from China. If we need these two kinds of data in our work, we can take some measures to obtain them from foreign databases [12]. ASTERGDEM, namely, GDEM-V1 and SRTMC can be downloaded from the Chinese Academy of Sciences. Most of the accuracy obtained here can meet the required requirements. Relatively speaking, the accuracy of the data obtained by GDEM-V1 is higher. It is difficult to obtain data from GMTED, and the accuracy of the obtained data is not high, so we basically do not use this method. Converting TIN to Grid in ArcToolbox will lay the foundation for our next terrain analysis [13]. Run the command TINtoGrid in 3DAnalyst to get the TinGrid data. Execute the surface analysis-slope command in 3DAnalyst, set the parameters to select the input surface as TinGrid and get the slope grid SlopofTinGrid. The slope is then reclassified. If it is the same grade, execute the surface analysis-aspect command in 3DAnalyst. For Qujiang New District of a certain city, the results of calculation from DEM topographic data are shown in Figure 3 for the analysis results of elevation, slope, and aspect of a certain city [14].

### 3.2. Propose a Water System Construction Method Suitable for the Terrain and Reasonable Overall Indicators

**Figure 4:**
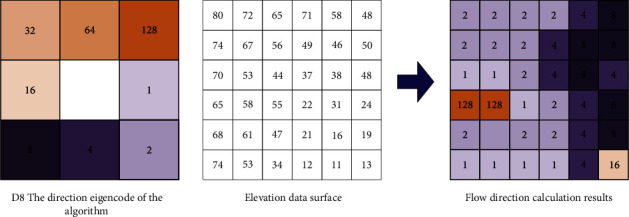
A generalized diagram of ArcGIS water flow direction D8 algorithm.

In ArcGIS, the direction of water flow is the basis for analysis using DEM elevation data. Judge the direction of the water flow in the grid we made, and the water flow has the law of flowing from a high place to a low place. In each small grid, the flow direction of water is also a process from high to low [15]. Now, there are two methods of single flow direction algorithm and multiple flow direction algorithm to determine the flow direction of water flow. Compared with the multiflow algorithm, the single-flow algorithm is simpler and more accurate, so we generally choose the simple and convenient single-flow algorithm when judging the water flow [16]. In Hydrology, the hydrology processing module in ArcGIS, the D8 method in the single flow direction algorithm is used to determine the flow direction of the water flow. Now, we use a grid information to illustrate that the flow of water flows in only 8 situations, namely, due east, due west, due south, due north, and southeast, southwest, northeast, and northwest; as shown in Figure 4 ArcGIS water flow direction D8 algorithm generalization diagram.

In ArcGIS, the maximum distance weight difference between the center grid and the adjacent grid is calculated to determine the flow direction. The process of determining the direction of water flow in ArcGIS is: open ArcToolbox in ArcMap, start the SpatialAnalysisTools module, find the Hydrology toolset, click on the FlowDirection tool, and the page for calculating the direction of water flow will appear. Provide the data type to the calculation software. The data type we want to discuss is DEM data, so enter the DEM data type, name the file and save it. Select the raster flow direction according to the DEM data provided by us, and it is not selected by default. The output drop raster is the output distance weight drop, expressed as a percentage; here we do not make a choice. In the simulated catchment path, the cumulative result is calculated based on the data of the water flow direction. This principle is based on the regular grid calculation of DEM data without depressions. We assume that each small grid has a unit volume of water flow. And, because the direction of the water flow is from high to low, we can roughly calculate the water flow through each grid, and finally calculate the value of the water flow of these grids.

The water flow direction is judged on the basis of the DEM data without depressions calculated above, the FlowAccumulation tool is used in the Hydrology tool set, and the regional confluence accumulation can be obtained through the relevant data of the water flow direction obtained. A drainage basin refers to a concentrated area formed by the discharge of water or pollutants from a certain area. From the basin data, we can know the size of the catchment area within the scope of our study. Generally speaking, the place where water flows out of the basin is the lowest point in this area. The watershed basin is bounded by watersheds, and the area surrounded by watersheds becomes the catchment area. The watershed divides the entire watershed basin into multiple catchments. In ArcGIS, the water direction data is analyzed to calculate the relevant sinks. Water path, and count the amount of water from the same path. The first thing we need to do is to find the outlets of the catchment area and the small grid where all outlets are located.

Input the DEM elevation data into the algorithm to calculate the direction of water flow, and use the corresponding tool to get the natural catchment area. The use of Tyson polygons divides the natural catchment area into multiple partial catchment zones so that each outlet has a corresponding catchment zone. According to a city's urban planning and the characteristics of water catchment area division, it is judged as three levels. A comprehensive analysis of the characteristics of a certain city's watershed and water system determines the first-level division; on the basis of the first-level division, the second-level division combines the control planning unit and the watershed division; the third-level division proposes methods to determine the control indicators of each district. According to the development index of sponge city shadow, it is also divided into three levels. The first level is the data in the divided first level partition, the second level is the data in the divided second level partition, and the third level should consider the specific conditions and determine the corresponding indicators according to the actual situation. Combined with the classification and structure of the underlying surface construction, land development, etc., the weighted average method is used for calculation. The ability of each area to build sponge cities based on the sponge city construction status, green area ratio, water surface ratio, construction intensity, and other factors in each district are evaluated. First, obtain the total annual runoff control rate of each district and use the weighted average method to calculate and determine the total annual runoff of the city. The volume control rate meets the requirements of urban planning, construction, and management control. If the requirements are not met, the total meridian flow control rate of each district should be adjusted according to the planned land use situation, underlying surface construction intensity and hydrogeological characteristics, and finally reach the control range. In order to evaluate the ability of each district to build a sponge city and whether it has the conditions for building a sponge city, this article first analyzes and counts the status quo of the first-level districts and analyzes the proportion of built-up areas, green area ratios, and water surface ratios of the first-level districts. Quantitative scoring on 1–5 levels. Evaluation factors for the suitability of sponge city construction in the secondary zone is as shown in Table 1.

Then, through the corresponding scores of the green space rate, water surface rate, and built-up area ratio of each district and then assign corresponding weights to them according to their importance in reality and then obtain the total score of each area, comprehensively evaluate the contribution of each district to the construction of sponge cities. The ability and whether it can be built into a sponge city.

(1) $$\begin{array}{c} Comprehensive\;score=\left(Green\;Space\times 3+Surface\;rate\times 3+Completion\;zone\;ratings\times 4\right)/10\text{.} \end{array}$$

### 3.3. Sponge Tissue Method Combined with Natural Topography

This section first explains how to construct a sponge city system based on topographical conditions. It also refers to the domestic and foreign control and operation methods of rainfall runoff and what technical means they use to operate and then compares my country's topographic factors and construction of sponges. For the conditions required by the city, construct a topographic feature index and comparison benchmark model, as shown in Table 2 Applicable Topographic Feature Index Evaluation and Comparison Benchmark for Sponge Facilities.

### 3.4. Check the Layout of Sponge Facilities

Sponge city planning and construction are often related to newly-built areas and old urban areas. Compared with newly-built areas, the conflicts of interest in old urban areas are relatively complex and the degree of development is relatively large. For this mature area, the layout of sponge facilities is of special significance. Even in low-lying areas with relatively poor soil permeability, it is not suitable to take up too much area for water storage facilities such as ponds. At this time, it is necessary to adjust the selection of sponge facilities according to local conditions and measure the function and scale of various facilities according to the water distribution index and economic value in the area. For example, consider whether it is possible to use sponges to cover all green areas or place some water storage tanks underground to offset some of the indicators, or whether to increase the area of green roofs to purify rainwater and to infuse and store the purified rainwater. Biodiversity is threatened by various road networks, which has attracted the attention of many scholars at home and abroad who design safe passages for wildlife. In the arrangement of sponge facilities, if there is a conflict between wetland parks and urban highways, it should be considered whether motor vehicles pose a threat to the lives of animals. We should make use of reasonable time intervals and take some protective measures for wild animals in appropriate places to provide a guarantee for the living environment and life safety of wild animals.

**Figure 5:**
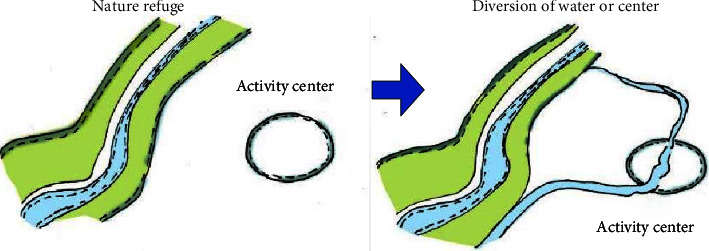
Combining sponge facilities with activity space to enrich the landscape.

Combining sponge facilities with other landscape facilities, consider whether there is a connection between landscape facilities and sponge facilities from multiple aspects. When the beach is distributed near the landscape facility, the water system can be introduced into the sponge facility. The introduction of sponge city facilities can not only enhance the ability to store rainwater but also bring people a bright visual enjoyment, enabling them to realize the function of playing a role and enriching the landscape. As shown in Figure 5, the sponge facilities and the activity space are combined to enrich the landscape.

When the development of the city affects the construction of the sponge city, we can make reasonable use of the sponge facilities to achieve the indicator of rainwater control. If the rainwater purification facilities we want to achieve cannot be achieved, we can increase the green area of the roof and rationally design rainwater purification facilities to achieve the purpose of rainwater purification and storage. For another example, if the sunken green space reduces the survival rate of plants due to some objective conditions, it is difficult to use the rainwater that seeps into the ground, we can reprocess the sponge facilities.

We should first fully understand the local terrain and the functions of sponge facilities and make a reasonable plan and then start to implement it. However, we need to know that the terrain is not the only criterion for testing whether the layout of the sponge facilities is reasonable. Need specific analysis. Arranging sponge facilities is not the only way to solve related problems. We can design or even transform the function and scale of sponge facilities to meet the required requirements. When we select the location of sponge facilities, we should comprehensively consider the local natural topography, building conditions, development conditions, etc. In order to reduce the occurrence of disasters, it is also an indispensable step to check whether there are earthquake zones in the layout area. Consider the influence of setting sponge facilities on various factors before layout.

## 4. GIS-Based Urban Information System Development Method

### 4.1. System Design Pattern

After more than 30 years of development and practical application of GIS, there has been a fairly mature system including a software cycle model and a rapid prototyping model. In the actual development process, these two models are often combined and applied. The rapid prototyping model is used to sort out the requirements, and the software cycle model is used to carry out the subsequent work to ensure the orderly progress of the work. The rapid prototyping model is mainly a model that enables the software system to run quickly through rough analysis. By solving the problems feedback from users, continuous improvement, and finally achieve the results that are satisfactory to both parties. Compared with the traditional method, the object-oriented method has more advantages. Of course, the object-oriented method is not a method without any defects. The object-oriented method absorbs the essence of structured thinking and supplements the structured thinking.

The urban planning and management information system platform is a large-scale software consisting of data engineering, software engineering, and hardware engineering. What is interesting is that software engineering also includes hardware.  Table 3 shows the key issues and main tasks of each work stage.

For the planning and management of urban construction, the platform software uses geographic information (GIS), office automation (oA), computer networks, and other technical means to achieve comprehensive applications such as homework, data sorting, and image processing. According to consistent information classification standards, a series of data generated during the planning and management of the city are managed in a unified manner to obtain a shareable resource. Relevant staff can check the authenticity of these data anytime and anywhere, and some new approval information and opinions can also be included in the database in a timely manner. According to the work tasks and work processes of the staff of each department, the corresponding registration, inquiry, and statistical processes are proposed and applied to realize the management and supervision process achieved through the computer network.

### 4.2. System Structure

**Figure 6:**
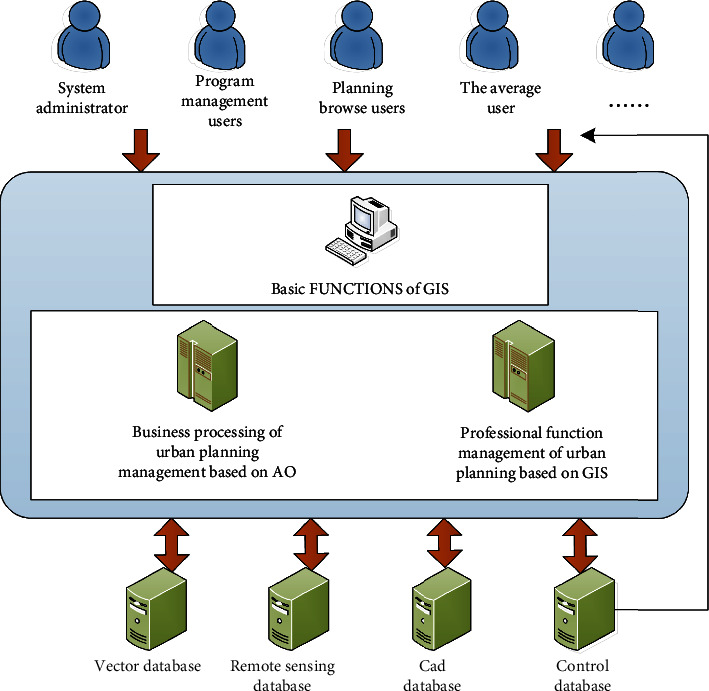
Platform architecture.

The basic functions of this platform are provided by a series of GIS functions, and multiple modules are developed according to management requirements. At the same time, the platform has installed an automation module in the platform in order to reduce the work pressure of the staff in the planning department. It can be seen from Figure 6 that after the user logs in to the platform software, he uses the software's functions to call the database to achieve the purpose of database management.

The functions of the software development platform are divided into the following parts: user authority management; basic GIS functions; GIS functions related to urban management and control work; OA functions related to urban management and control work. On top of this, break it down into modules capable of different data encapsulation functions, so that they can further form components. Only the necessary data interfaces are disclosed to the outside world; as long as the required standards are met, the program can be inserted into the platform software, all of which greatly improve the scalability of the platform. In the process of urban management and control, the platform software widely uses component development methods.

## 5. Conclusion

This article discusses the influence of natural topography on the planning and construction of sponge cities, including whether natural topography can affect rainwater, whether it can affect the distribution of different grounds, whether it can affect the use of groundwater resources, and whether it is because of this topography. In the event of a natural disaster, if you think that changing the topography will have any impact on it, then analyze whether the stability of the natural topography of the northern cities is affected by rain. At present, the situation of informatization construction is developing rapidly. Most of the relevant personnel in the urban planning work departments have begun to build their own information systems. However, it seems that the established systems are almost only office automation systems. A small number of geographic information systems and office automation systems are combined, and the comprehensive application of G1S, OA, and MIS is rarely achieved. The software platform design process proposed in this article strives to reflect the perfect combination of the three methods, and in the software platform, the idea of workflow is realized in the components of, users can independently view the working status and perform operations, which provides convenient conditions for urban management and control staff. This article combines VB and ArcGISEngine to build platform development software. This software has the characteristics of easy operation, simple page, and convenient and quick user use.

## Figures and Tables

**Table 1:** Evaluation factors for the suitability of sponge city construction in the secondary zone.

Factor	Range	Scoring
Green area rate	<20%	1
	20%∼30%	2
	30%10%	3
	40%∼50%	4
	>50%	5

Surface rate	<3%	1
	3%∼6%	2
	6%∼9%	3
	9%∼12%	4
	>12%	5

Proportion of built-up area	>85%	1
	60%∼85%	2
	45%∼60%	3
	20%∼45%	4
	<20%	5

**Table 2:** Applicable topographic feature index evaluation and comparison criteria for sponge facilities.

Sponge facility		Location characteristics		Natural terrain conditions					
				Soil condition	Groundwater characteristics	Topography	Nature of catchment		Space requirement
		Land use type	Pollution load intensity	Soil type	The highest underground water level is from the bottom of the facility (m)	Confluence gradient (%)	Catchment area (ha)	Impermeability (%)	Area (ha)
Permeable sponge facilities “seepage”	Permeable paving	R/B/G/S	Low	A-B	>0.61	1–3	<1	>0	—
	Sunken green space	R/S/B/G	In	A-B	>0.61	1–5	>4	>0	In
	Biological retention facility	R/S/B/G	In	A-B	>1.22	1–5	<4	>0	In
	Infiltration pond	R/S/B/G	In	A-B	>3	<15	1–4	>0	Big
	Seepage well	R/S/B/G	Low	A-B	>0.61	<10	<1	>0	Small

Adjustment category sponge facilities “stay”	Regulating pond	R/G	Low	A-D	>1	<10	>6	>0	Big

Adjustment category sponge facilities “stay”	Regulation pool	R/S/B/G/M	Low	A-D	>1	—	—	>0	Small

Storage type sponge facilities “fan, use”	Wet pond	R/G	High	A-D	>1.22	<10	>6	0–80	Big
	Rain wetland	R/G	High	B-D	>1.22	4–15	>10	0–80	Big
	Reservoir	R/S/B/G	Low	A-D		—	—	>0	Small
	Rainwater irrigation	R/B	Low	—	—	—	—	—	Small

Purification category sponge facility “clean”	Green roof	R/B/M	In	—	—	<4	—	—	—
	Vegetation buffer zone	R/S/B/G/M	High	A-D	>0.61	2–6	>4	>0	In
	Initial rainwater abandonment facility	R/B/M	High	—	—	—	—	—	Small
	Artificial soil	R/B/M	In	A-D	>0.61	<10	<2	0–50	Small

Transfer type sponge facility “row”	Zhicaogou	R/S/B/G	In	A-D	>0.61	0.5–5	<2	>0	In
	Seepage pipe/drain	R/G	Low	A-B	>1.22	—	<2	>0	In

**Table 3:** Key issues and main tasks of each work stage.

Work phase	The key issue	Main mission
Definition and plan	What is the question?	Determine the outline of the system plan; survey tasks for the transportation requirements; make preparations for the survey
Feasibility study	Is there a feasible solution?	Provide expert opinions and suggestions
Demand analysis	What must the system do?	Understand the artificial system model; determine user needs; order current business flow and data flow; collect original business data, analyze demand survey results, and make design preparations
Outline design	How to solve the problem?	Determine system design principles and goals, carry out system control structure design, software function structure design, database design, operating environment design and interface design; determine system implementation plan
Detailed design	How to implement the system concretely?	Familiar with the basic development platform software; determine the module function and the calling relationship between the modules; determine the module interface; give the module control mix diagram 1 gives the software guide and user input and output interface
Program code	How to design the correct program unit?	According to detailed design results and programming guidelines, the functions of each caution block are realized with code
Test inheritance	How to ensure that the software meets the requirements?	Install and develop the application system on-site, and conduct anti-image debugging through simulation operation; meanwhile, conduct system use training for business personnel at all levels of the planning bureau
Operation and maintenance	How to make the system persistently meet the requirements?	The system enters the formal operation stage, during which system identification is completed; operation and maintenance are carried out

## Data Availability

The data used to support the ﬁndings of this study are available from the corresponding author upon request.
